# Total Body Water and Fluid Distribution in Relation to Musculoskeletal Injury History: A Bayesian Analysis in Physically Active Young Adults

**DOI:** 10.3390/jcm15218392

**Published:** 2026-10-28

**Authors:** Jarosław Domaradzki

**Affiliations:** Department of Biological Principles of Physical Activity, Wroclaw University of Health and Sport Sciences, 51-612 Wrocław, Poland; jaroslaw.domaradzki@awf.wroc.pl

**Keywords:** total body water, extracellular water, fluid distribution, musculoskeletal injury, sports injury, Bayesian analysis, physical activity, training exposure

## Abstract

**Background/Objectives:** Body water status is relevant to physiological function during physical activity, yet its relationship with musculoskeletal injury occurrence remains insufficiently understood. This study examined whether total body water (TBW) and relative extracellular water distribution (ECW/TBW) were associated with injury occurrence in physically active young adults using Bayesian inference. **Methods:** Analysis included 418 physically active university students (199 men, 219 women). Body-water characteristics were measured at a single time point, whereas musculoskeletal injuries were retrospectively reported for the preceding 12 months. Bayesian logistic regression models examined associations of TBW and ECW/TBW with injury history, adjusting for sex, total physical activity, weekly training volume, and training age. Posterior probabilities were evaluated for meaningfully negative, practically negligible, and meaningfully positive associations using a predefined region of practical equivalence (ROPE; β = −0.10 to +0.10). **Results:** Overall, 51.4% of participants reported at least one injury. Higher TBW was associated with higher odds of reporting an injury in the adjusted model (OR = 1.38, 95% credible interval [CrI]: 1.11–1.73), with a 97.8% posterior probability of a meaningfully positive association. In contrast, higher ECW/TBW was associated with lower odds of injury occurrence (OR = 0.77, 95% CrI: 0.62–0.95), with a 93.6% posterior probability of a meaningfully negative association. The direction of both associations remained stable across unadjusted models and alternative ROPE definitions. **Conclusions:** TBW and relative extracellular water distribution showed distinct and opposing associations with retrospectively reported musculoskeletal injury history. These findings provide information about cross-sectional relationships but do not establish temporal direction or prospective injury risk. Longitudinal studies are needed to clarify their physiological and potential predictive relevance.

## 1. Introduction

Musculoskeletal injuries are common among physically active young adults and may interrupt training, reduce participation in physical activity, and adversely affect subsequent physical functioning [1,2]. Their occurrence is multifactorial and reflects the interaction between exposure to physical activity or training and individual characteristics that influence the ability to tolerate mechanical and physiological demands [3,4,5]. Accordingly, injury research has considered a broad range of potential determinants, including previous injury, training load, physical fitness, body composition, and demographic characteristics [6,7]. However, body composition is commonly described in terms of body mass, fat mass, or fat-free mass, whereas the water component of the body and its distribution between fluid compartments have received considerably less attention in the context of musculoskeletal injury [8,9].

Body water is integral to physiological function during exercise and contributes to thermoregulation, cardiovascular stability, electrolyte balance, substrate and metabolite transport, and normal neuromuscular function [10]. Alterations in hydration can affect exercise capacity, perceived exertion, and physiological responses to physical stress, providing a plausible link between fluid status and the conditions under which injuries may occur [11]. Most available evidence, however, has approached this issue from the perspective of acute hydration or dehydration, particularly in relation to exercise performance, heat stress, and fatigue [12,13]. Acute changes in hydration status should be distinguished from interindividual differences in body water characteristics measured under habitual conditions. The latter may reflect not only current fluid availability but also differences in body composition and the relative distribution of water within the body, potentially providing a broader physiological characterization of physically active individuals [14].

From this perspective, both the overall amount and the compartmental distribution of body water may be informative. Total body water (TBW), expressed relative to body mass, characterizes the overall water component of the body, whereas the extracellular water-to-total body water ratio (ECW/TBW) describes the relative contribution of extracellular water to total body water [15]. These measures therefore represent related but conceptually distinct aspects of body water status: individuals may differ in overall relative water content as well as in how that water is distributed between intracellular and extracellular compartments [16]. Because intracellular and extracellular water proportions are complementary components of TBW, their simultaneous consideration as independent variables associated with injury history would provide largely reciprocal information. Evaluating TBW together with ECW/TBW may therefore offer a parsimonious approach to distinguishing overall body water content from its relative compartmental distribution when examining their relationships with musculoskeletal injury occurrence [8].

Despite the established physiological importance of hydration, little is known about whether interindividual differences in total body water and its compartmental distribution are associated with musculoskeletal injury occurrence. Previous research has focused primarily on acute hydration and dehydration, whereas habitual body water characteristics have received considerably less attention in the context of injury [17,18]. Examining both overall body water and its relative distribution may provide complementary information on physiological characteristics associated with injury occurrence, particularly when differences in physical activity and training exposure are considered. The present study therefore examined TBW and ECW/TBW as complementary indicators within a Bayesian framework that quantified posterior support for meaningfully negative, practically negligible, and meaningfully positive associations.

The present study uses a pooled dataset that has also been examined in previous publications addressing other research questions, including lifestyle behaviors, dietary patterns, body composition, and musculoskeletal injury characteristics. However, these earlier analyses did not specifically investigate total body water and relative extracellular water distribution as separate correlates of retrospectively reported musculoskeletal injury history. Furthermore, the present study applies a Bayesian logistic regression framework to quantify posterior support for negative, practically negligible, and positive associations, while accounting for sex, physical activity, and training exposure. Thus, its contribution lies in the examination of previously unexplored body-water indicators in relation to injury history and in the probabilistic characterization of their associations, rather than in the introduction of a new study population.

The present study aimed to investigate the relationships of total body water and relative extracellular water distribution with sports-related musculoskeletal injury occurrence among physically active young adults, adjusting for sex, total physical activity, weekly training volume, and training age. Specifically, the study addressed the following questions: (1) Does total body water show evidence of a meaningfully negative, practically negligible, or meaningfully positive association with injury occurrence? (2) Does the relative contribution of extracellular water to total body water (ECW/TBW) show evidence of a meaningfully negative, practically negligible, or meaningfully positive association with injury occurrence? (3) Do these posterior conclusions remain consistent after adjustment for sex, total physical activity, weekly training volume, and training age? Finally, sensitivity analyses examined whether the conclusions were robust to alternative definitions of the region of practical equivalence.

## 2. Materials and Methods

Data were pooled from two cohorts of physically active university students who underwent assessments in 2022 and 2023 according to an identical study protocol. Only participants with complete data required for the present analyses were included.

### 2.1. Study Design

The study employed a cross-sectional approach, with injury history collected retrospectively for a specified recall period. A convenience sample was drawn from students enrolled in physical education, sport, and physiotherapy programs at the Wroclaw University of Health and Sport Sciences.

Data collection involved an online questionnaire addressing previous injuries, physical activity, and training-related characteristics, followed by laboratory-based assessments of anthropometric parameters and body composition.

### 2.2. Ethics

Ethical approval was granted by the Senate Research Ethics Committee of the Wroclaw University of Health and Sport Sciences (approval no. 13/2022). The research adhered to the principles of the Declaration of Helsinki, and electronic informed consent was obtained from all participants prior to data collection.

### 2.3. Sample Size

An a priori sample-size calculation was based on a standard proportion formula [19]. Assuming a 95% confidence level, a 5% margin of error, and a conservative expected proportion of 0.50, the minimum sample size was 385 participants. Allowing for incomplete data, the recruitment target was increased by 20% to approximately 460 participants.

### 2.4. Participants

**Figure 1:**
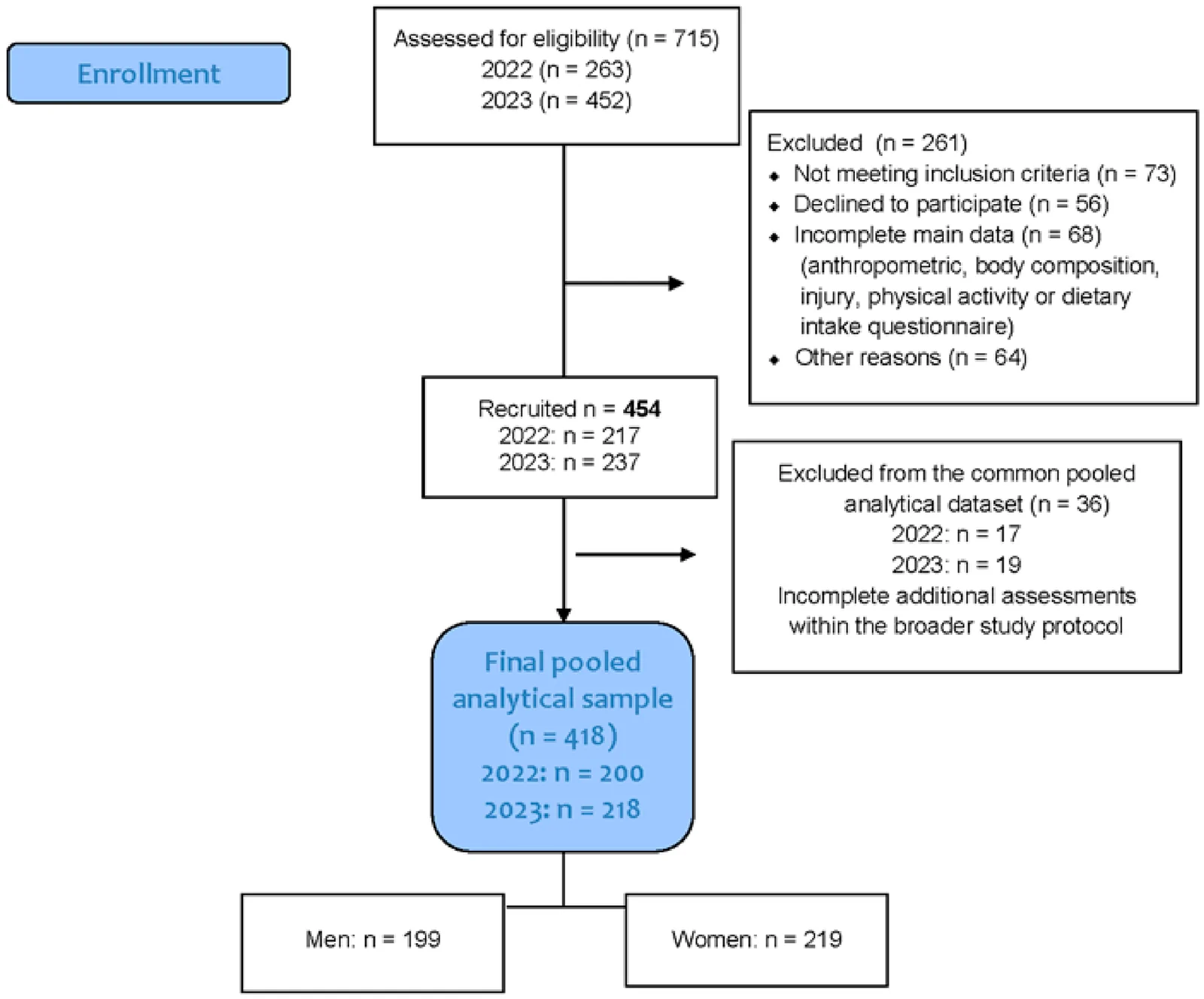
Flowchart of participant recruitment, exclusions, and derivation of the final pooled analytical sample across the 2022 and 2023 cohorts.

A total of 454 students were recruited across two cohorts (2022: *n* = 217; 2023: *n* = 237). Of these, 36 participants (2022: *n* = 17; 2023: *n* = 19) were excluded from the common pooled analytical dataset because of incomplete additional assessments within the broader research protocol, including sleep-related questionnaires, balance measurements, and other assessments not examined in the present study. The final analytical sample comprised 418 participants (2022: *n* = 200; 2023: *n* = 218), including 199 men and 219 women. This predefined pooled sample was retained to maintain consistency across related investigations addressing distinct research questions. Participant selection is illustrated in Figure 1.

### 2.5. Anthropometric Measurements

Anthropometric and body composition assessments were conducted at the Biokinetics Research Laboratory, Wroclaw University of Health and Sport Sciences. Standing height was measured to the nearest 0.1 cm using a GPM anthropometer (GPM Instruments GmbH, Susten, Switzerland), whereas body mass and composition were assessed using a bioelectrical impedance analyzer (InBody 230, InBody Co., Ltd., Seoul, Republic of Korea). Body mass index (BMI) was calculated as body mass (kg) divided by squared height (m^2^). Body composition assessments included fat mass, fat-free mass, and body-water compartments. The present study focused specifically on total body water (TBW), intracellular water (ICW), and extracellular water (ECW), expressed as percentages of body mass.

All body composition measurements were conducted in the morning, between 7:30 and 8:30 a.m., before scheduled university classes. Participants were instructed to refrain from eating before the assessment and to avoid fluid intake for at least one hour prior to measurement. Compliance with these recommendations was not independently verified. Measurements were performed barefoot, with participants wearing sports clothing.

### 2.6. Questionnaire Measurements

#### 2.6.1. Injury Occurrence—Injury History Questionnaire (IHQ)

Information on musculoskeletal injuries sustained over the previous 12 months was collected through the Injury History Questionnaire (IHQ) [20]. Participants reported injuries sustained during sport, recreational, or daily activities. The primary outcome included musculoskeletal injuries regardless of the activity context in which they occurred; sports-related and non-sports-related injuries were not distinguished in the regression analyses. No minimum injury severity threshold, such as a specified period of activity restriction or a requirement for medical attention, was applied. For the present analyses, injury history was coded as a binary variable (0 = no injury; 1 = ≥1 injury), and the total number of injuries was retained as a count variable. Thus, the primary outcome represented retrospectively reported musculoskeletal injury history rather than prospectively recorded sports injury incidence.

#### 2.6.2. Physical Activity (PA)—International Physical Activity Questionnaire (IPAQ)

Physical activity (PA) levels were quantified using the Polish adaptation of the International Physical Activity Questionnaire—Long Form (IPAQ-LF) [21], with total activity expressed in MET·min/week.

#### 2.6.3. Training Characteristics—Training Volume (TV) and Training Age (TA)

Training volume (TV) was quantified as the number of hours devoted to sport-specific training each week [22]. Training age (TA) reflected the total duration of systematic participation in sport-specific training, measured in years [23].

### 2.7. Statistics

The analytical strategy was designed to evaluate evidence for associations between body water status and musculoskeletal injury occurrence, progressing from descriptive characterization of the sample to Bayesian modeling and evaluation of posterior evidence for negative, practically negligible, and positive associations.

#### 2.7.1. Descriptive Analyses

Descriptive analyses were performed for the entire study population, with additional stratification by sex and injury status where applicable. Continuous data were presented using means and standard deviations or medians and interquartile ranges, depending on their distributions. Categorical data were summarized by absolute frequencies and percentages. Body water characteristics included total body water (TBW) and the relative distribution of intracellular and extracellular water (ICW/TBW and ECW/TBW). Between-group comparisons were considered descriptive and were not used to determine variable inclusion in the Bayesian models.

#### 2.7.2. Primary Body Water Indicators

TBW and ECW/TBW were selected as physiologically distinct indicators for inferential analyses, representing overall body water relative to body mass and the proportion of total body water contained in the extracellular compartment, respectively. The ECW/TBW and ICW/TBW ratios were calculated by dividing the respective compartment values by TBW and were expressed as percentages for descriptive purposes. ICW/TBW was retained for descriptive characterization but was not modeled separately because the two compartment ratios provide essentially reciprocal information. TBW and ECW/TBW were analyzed in separate Bayesian logistic regression models, with both predictors standardized prior to analysis.

#### 2.7.3. Bayesian Regression Analyses

Bayesian logistic regression constituted the primary inferential framework. Injury history was defined as a binary outcome (0 = no reported injury; 1 = at least one reported injury during the preceding 12 months). Separate models were fitted for TBW and ECW/TBW. Each body-water indicator was examined using two models: an unadjusted model assessing its association with injury history and a subsequent model adjusted for sex, total physical activity, weekly training volume, and training age. These covariates were specified a priori on substantive grounds to account for differences in physical activity and training exposure, rather than being selected according to statistical significance in descriptive comparisons.

Continuous predictors were standardized before modeling so that regression coefficients represented the expected change in log-odds of reporting an injury per 1-SD increase in the respective predictor. Bayesian logistic regression models were fitted using the brms package in R, with a Bernoulli likelihood and logit link function. Weakly informative normal priors were assigned to regression coefficients (Normal(0, 1)) and the intercept (Normal(0, 1.5)). Each model was estimated using four Markov chain Monte Carlo chains with 4000 iterations per chain, including 1000 warm-up iterations, yielding 12,000 post-warm-up draws in total. The target acceptance probability (adapt_delta) was set to 0.95, and the random seed was fixed at 1234.

**Figure 3:**
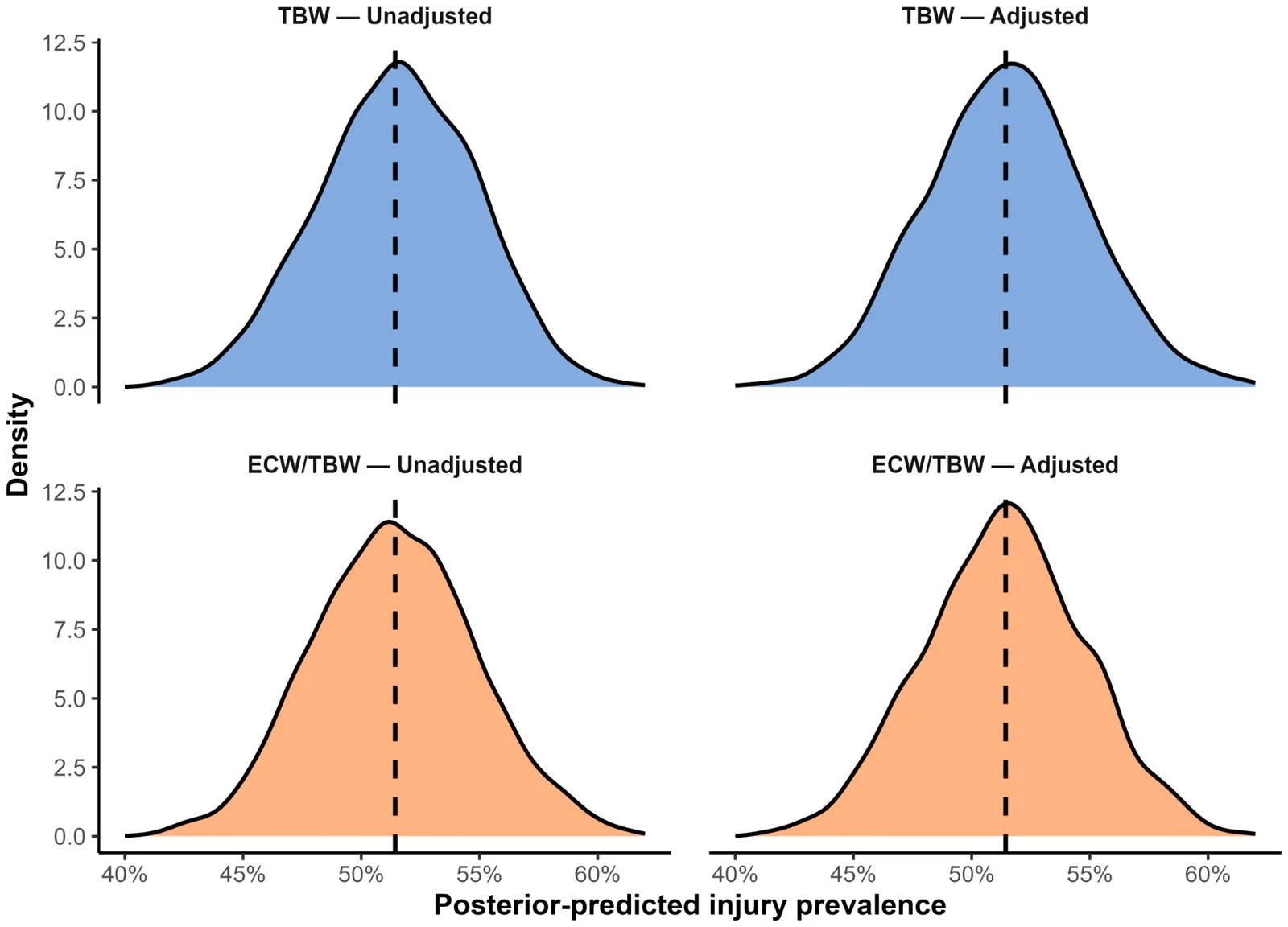
Posterior predictive checks for the Bayesian logistic regression models of injury occurrence. Distributions represent posterior-predicted proportions of participants reporting at least one musculoskeletal injury for the unadjusted and adjusted models of total body water (TBW) and extracellular water relative to total body water (ECW/TBW). The dashed vertical line indicates the observed injury prevalence (51.4%). Adjusted models included sex, total physical activity, weekly training volume, and training age.

Convergence and sampling diagnostics from an independent Python MCMC analysis are provided as a supplementary numerical check in Supplementary Table S1. These diagnostics do not represent the original brms/Stan sampling diagnostics. Posterior predictive checks were used to compare observed and model-generated injury prevalence and are presented in Figure 3.

#### 2.7.4. Evaluation of Evidence for Negative, Negligible, and Positive Associations

The Bayesian framework was used to quantify the relative posterior support for three competing interpretations of each association. A region of practical equivalence (ROPE) from −0.10 to +0.10 on the standardized log-odds coefficient scale was specified as the primary reference interval. This corresponds to odds ratios ranging approximately from 0.90 to 1.11 per 1-SD increase in the predictor, representing relatively small associations with retrospectively reported injury history. The interval was adopted as an operational threshold for distinguishing small associations from potentially meaningful directional effects, rather than as an established clinically validated threshold for musculoskeletal injury outcomes. Because no universally accepted minimum clinically important association has been established for these body-water indicators in relation to injury history, sensitivity analyses were conducted using narrower (±0.05) and wider (±0.20) ROPE definitions to assess the dependence of posterior conclusions on this choice.

Posterior distributions were partitioned into three regions: a meaningfully negative association (H−: β < −0.10), a practically negligible association (H0: −0.10 ≤ β ≤ 0.10), and a meaningfully positive association (H+: β > 0.10). For each parameter, the corresponding posterior probabilities, P(H−|data), P(H0|data), and P(H+|data), were calculated. This approach allowed the relative evidence for each of the three possibilities, including practical equivalence, to be quantified directly.

Posterior regression coefficients are reported with 95% credible intervals (CrIs) and the corresponding posterior probabilities for H−, H0, and H+. Regression coefficients were additionally exponentiated and reported as odds ratios (ORs) with 95% CrIs.

#### 2.7.5. Model Evaluation and Sensitivity Analyses

Model adequacy was evaluated using MCMC convergence diagnostics and posterior predictive checks, as described in Section 2.7.3. Sensitivity analyses examined the robustness of posterior conclusions to alternative definitions of practical equivalence and prior specifications. The ROPE sensitivity analysis considered thresholds of ±0.05 and ±0.20 in addition to the prespecified reference threshold of ±0.10. Prior sensitivity was assessed using more strongly regularizing (Normal(0, 0.5)) and more weakly regularizing (Normal(0, 2)) priors for regression coefficients, compared with the primary Normal(0, 1) prior. The intercept prior was retained at Normal(0, 1.5). For the additional prior specifications, posterior distributions were evaluated using Laplace-based importance sampling, and posterior means, 95% credible intervals, and probabilities of the three directional hypotheses were compared across specifications.

An additional sensitivity analysis was conducted to examine potential confounding by body composition. Fat-free mass index (FFMI), calculated as fat-free mass divided by height squared (kg/m^2^), was added to the adjusted TBW and ECW/TBW models. FFMI was standardized before modeling. The resulting posterior estimates were compared with those from the corresponding primary adjusted models to assess whether the associations with injury history were sensitive to additional adjustment for fat-free mass relative to height.

All analyses were conducted in R/RStudio (version 2025.09), primarily using the brms package for Bayesian model estimation. Descriptive analyses were additionally performed, where appropriate, in Statistica 14.0 (TIBCO Software Inc., Palo Alto, CA, USA). Bayesian inference was based primarily on posterior distributions, credible intervals, and posterior hypothesis probabilities rather than conventional null-hypothesis significance testing.

In addition to posterior predictive checks of overall injury prevalence, model fit was further examined using the Brier score, the area under the receiver operating characteristic curve (ROC AUC), and comparisons of observed and predicted injury proportions across quintiles of fitted probabilities. These assessments were conducted within the analytical sample and were intended to characterize model fit rather than establish out-of-sample predictive performance.

### 2.8. Missing Data Handling

The present analysis was based on the common pooled dataset of 418 participants established within the broader research protocol (Figure 1). The exclusion of 36 participants was related to incomplete additional assessments not included in the present investigation. All participants retained in the analytical sample had complete data for the variables included in the Bayesian regression models, including injury history, TBW, ECW/TBW, sex, total physical activity, weekly training volume, and training age. The Bayesian regression models were fitted directly to the final analytical dataset of 418 participants. Multiple imputed datasets were not separately incorporated into the Bayesian modeling procedure, and no pooling of regression estimates across imputed datasets was performed.

### 2.9. AI Transparency Statement

The use of generative AI tools was restricted to the literature exploration, editorial support, and technical assistance, consistent with COPE and MDPI recommendations on transparency. Chat Academia (v.1.0, 2025), Elicit (v.2.0, 2025), and SciSpace (2025) supported the literature searches and manuscript preparation, while Julius AI (2025) and ChatGPT (OpenAI (San Francisco, CA, USA), v. GPT-6.0, 2026) assisted with language refinement and R code development and troubleshooting. No AI tools were employed to collect research data or independently interpret the study results. The author critically evaluated and verified all AI-assisted text and code and retains full responsibility for the statistical analyses and the final version of the manuscript.

## 3. Results

### 3.1. General Characteristics of the Study Sample

Table 1 presents the baseline characteristics of the study participants, with data expressed as means ± standard deviations and corresponding 95% confidence intervals. Data for age, body height, body weight, BMI, training weekly volume and training age have been presented previously [4]. Males had higher body size and fat-free mass indices than females (all *p* < 0.05). Body water measures also differed by sex, with males showing higher TBW, ICW, and ECW percentages than females (all *p* < 0.001). Men exhibited higher total physical activity (TPA) and longer training age (TA) than women (both *p* < 0.001), whereas weekly training volume (TV) did not differ significantly between sexes (*p* = 0.200).

Males also reported higher total physical activity (3608.2 ± 1356.9 vs. 3019.8 ± 1000.9 MET·min/week, *p* < 0.001) and longer training experience (3.61 ± 1.29 vs. 3.11 ± 1.47 years, *p* < 0.001). Weekly training volume did not differ significantly between males and females (6.14 ± 3.88 vs. 5.66 ± 3.82 h/week, *p* = 0.200). Detailed training and sport-participation characteristics of this cohort have been reported previously [24].

Of the 418 participants, 51.4% reported experiencing at least one musculoskeletal injury during the previous 12 months. The proportion of participants with an injury history was higher among men (56.8%) than women (46.6%), with a statistically significant difference between sexes (χ^2^ = 4.35, *p* = 0.037). Detailed characteristics of injury type, anatomical location, and recurrence in this cohort have been reported previously [24]. The present analyses therefore focused on injury occurrence (0/1) and the total number of reported injuries.

### 3.2. Bayesian Analysis of Injury Occurrence

Bayesian logistic regression indicated a positive association between TBW and injury occurrence (Table 2). In the unadjusted model, a 1-SD increase in TBW was associated with higher odds of reporting at least one injury (OR = 1.41, 95% CrI: 1.17–1.73). The posterior probability of a meaningfully positive association was 99.4%, whereas the probability of a practically negligible association was 0.6%. The association remained largely unchanged after adjustment for sex, total physical activity, weekly training volume, and training age (OR = 1.38, 95% CrI: 1.11–1.73). The posterior probabilities were 97.8% for a meaningfully positive association, 2.2% for a practically negligible association, and <0.1% for a meaningfully negative association.

An inverse relationship was observed for ECW/TBW. In the unadjusted model, each 1-SD increment in ECW/TBW corresponded to reduced odds of injury occurrence (OR = 0.76, 95% CrI: 0.62–0.92), with a 95.8% posterior probability of a meaningfully negative association and a 4.2% probability of a practically negligible association (Table 2). After covariate adjustment, the estimate remained similar (OR = 0.77, 95% CrI: 0.62–0.95), with posterior probabilities of 93.6% for a meaningfully negative association, 6.4% for a practically negligible association, and <0.1% for a meaningfully positive association.

Posterior predictive checks showed that the observed prevalence of musculoskeletal injury history was consistent with the distributions of injury prevalence generated by the fitted Bayesian models. Model-specific convergence and sampling diagnostics are presented in Supplementary Table S1.

**Figure 2:**
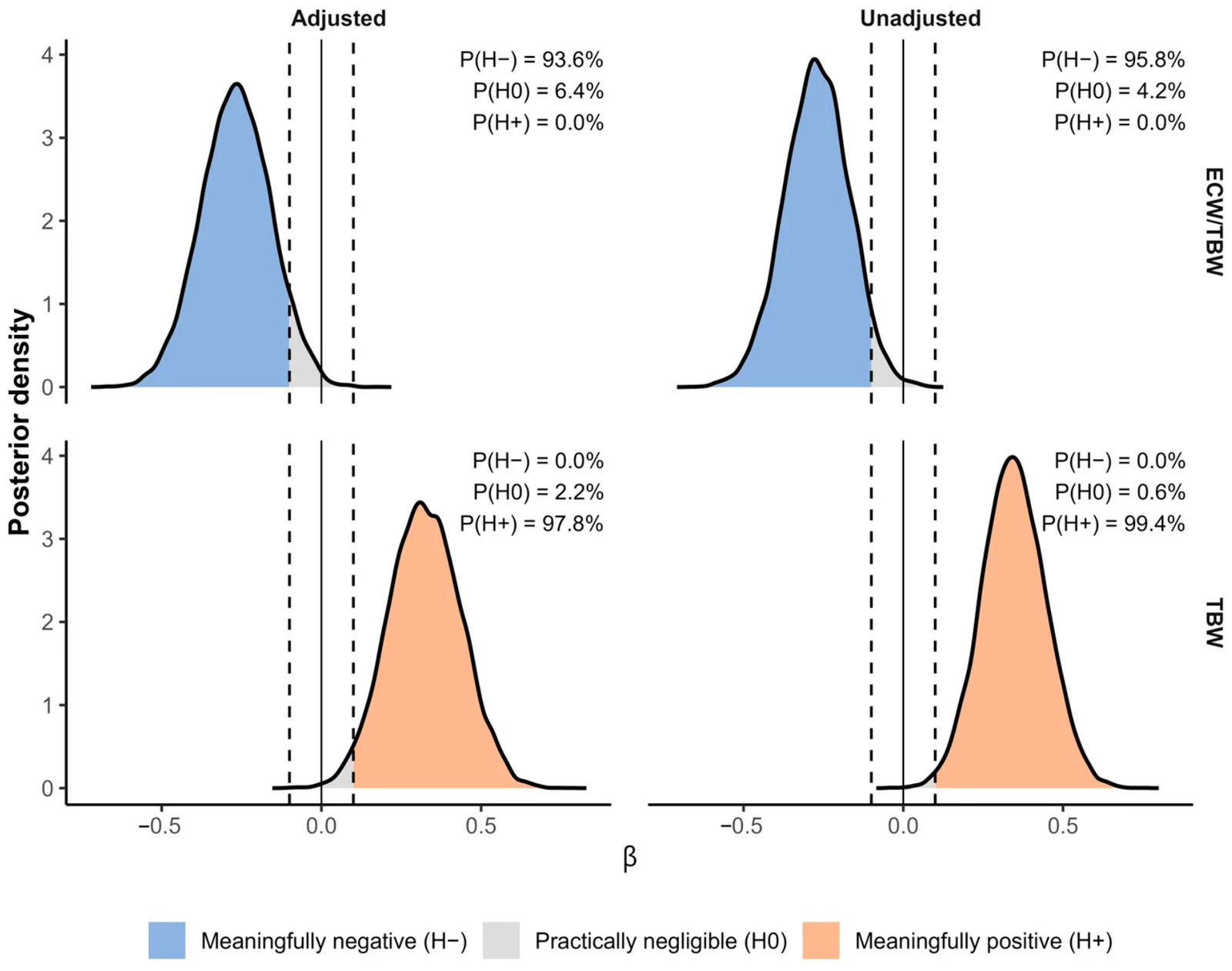
Posterior distributions of the associations between body water indicators and injury occurrence. Posterior distributions of standardized regression coefficients (β) are shown for total body water (TBW) and extracellular water relative to total body water (ECW/TBW) in unadjusted and adjusted Bayesian logistic regression models. The dashed vertical lines at β = −0.10 and β = +0.10 delimit the predefined region of practical equivalence (ROPE). The colored areas represent posterior support for a meaningfully negative association (H−; β < −0.10), a practically negligible association (H0; −0.10 ≤ β ≤ +0.10), and a meaningfully positive association (H+; β > +0.10). Posterior probabilities for the three regions are displayed within each panel. Adjusted models included sex, total physical activity, weekly training volume, and training age.

The posterior distributions and the corresponding probabilities of negative, practically negligible, and positive associations are presented in Figure 2.

Sensitivity analyses using narrower (±0.05) and wider (±0.20) ROPE definitions supported the robustness of the directional findings (Supplementary Table S2). In the adjusted models, the posterior probability of a meaningfully positive association for TBW ranged from 86.4% to 99.4%, whereas the probability of a meaningfully negative association for ECW/TBW ranged from 73.1% to 97.7%; posterior support for associations in the opposite direction remained negligible across all ROPE specifications.

### 3.3. Model Diagnostics and Sensitivity Analyses

The supplementary Python MCMC analysis showed R-hat values close to 1.00; approximate effective sample sizes are reported in Supplementary Table S1. Posterior predictive checks also indicated adequate model fit. The observed proportion of participants reporting at least one injury was 51.4%, and the mean posterior-predicted proportions were approximately 51.4% across all four models. The observed injury proportion was well within the corresponding 95% posterior predictive intervals, which ranged from approximately 44.5% to 58.4% across models (Figure 3).

Sensitivity analyses using alternative prior specifications yielded consistent results (Supplementary Table S3). In the adjusted TBW model, the posterior probability of a meaningfully positive association remained approximately 97.7% across all three prior specifications. Similarly, in the adjusted ECW/TBW model, the posterior probability of a meaningfully negative association ranged from 93.7% to 94.1%. Posterior effect estimates and credible intervals also showed only minor variation, indicating limited sensitivity of the observed associations to the prior distributions examined.

When TBW and ECW/TBW were included simultaneously in an additional model adjusted for the same covariates, the posterior mean coefficient for TBW remained positive, although uncertainty increased. The coefficient for ECW/TBW shifted toward zero, and both 95% credible intervals included zero (Supplementary Table S5).

Additional assessments of model fit indicated modest discrimination, with ROC AUC values ranging from approximately 0.58 to 0.61 across the four models. Brier scores ranged from 0.240 to 0.245, compared with 0.250 for the intercept-only reference prediction. Comparisons of observed and predicted injury proportions across quintiles of fitted probabilities showed some agreement but also revealed deviations in individual probability strata. These findings suggest that the models captured associations with injury history but had limited apparent discriminatory performance within the study sample.

### 3.4. Sensitivity Analyses Accounting for Fat-Free Mass Index

Additional sensitivity analyses were performed to examine whether the associations between body-water characteristics and retrospectively reported musculoskeletal injury history were influenced by differences in fat-free mass index (FFMI). After additional adjustment for FFMI, the positive association for TBW remained similar to that observed in the primary adjusted model (β = 0.306, 95% CrI: 0.087 to 0.527; OR = 1.359, 95% CrI: 1.090–1.693), with a posterior probability of 96.7% for a meaningfully positive association. Similarly, the negative association for ECW/TBW remained directionally consistent (β = −0.279, 95% CrI: −0.491 to −0.068; OR = 0.756, 95% CrI: 0.612–0.934), with a posterior probability of 95.1% for a meaningfully negative association. These preliminary sensitivity results suggest that additional adjustment for FFMI did not materially alter the estimated associations (Supplementary Table S4).

## 4. Discussion

This study investigated whether total body water and the relative distribution of extracellular water were related to musculoskeletal injury occurrence among physically active young adults. The findings revealed contrasting associations: higher TBW corresponded to greater odds of injury occurrence, with strong posterior evidence supporting a meaningfully positive association, while higher ECW/TBW was associated with lower odds of injury occurrence. Both associations remained similar after adjustment for sex, total physical activity, weekly training volume, and training age and were robust to alternative ROPE definitions. These findings suggest that overall body water content and its relative compartmental distribution may represent distinct characteristics associated with injury occurrence.

Although the present study draws on a previously analyzed cohort, its research question and analytical approach differ from those of earlier publications. Previous analyses examined broader lifestyle, body-composition, and injury-related characteristics, whereas the present investigation focuses specifically on TBW and ECW/TBW in relation to retrospectively reported injury history. The Bayesian framework additionally provides posterior probabilities for three predefined regions of association, allowing explicit evaluation of evidence compatible with negative, practically negligible, and positive effects. These findings should therefore be regarded as an extension of previous research using the same cohort, rather than as independent replication in a new sample.

Direct evidence linking body-water characteristics measured at assessment to musculoskeletal injury is limited, as previous research has focused predominantly on acute hydration, dehydration, exercise performance, and fatigue [12,13]. TBW should not, however, be interpreted simply as a marker of hydration, because it is strongly related to body composition, particularly water-rich fat-free tissue [25]. Previous research has also examined functional characteristics in relation to musculoskeletal injury history. For example, Gao et al. [26] investigated the association between previous sports injuries and lower-limb functional asymmetry in physically active university students. Although these findings concern functional performance rather than body composition, they illustrate that previous injury history may be associated with characteristics measured at the time of assessment. In a previous multidimensional analysis of the same cohort, body-composition characteristics showed statistically significant but generally weak relationships with injury phenotypes, with the clearest associations involving skeletal-muscle-related indicators and lower-limb injuries [24]. These findings suggest that body-composition characteristics may be related to previous musculoskeletal injuries, although the observed relationships were generally modest and do not establish prospective injury susceptibility. The persistence of the TBW association after adjustment for physical activity and training characteristics suggests that measured differences in training exposure did not fully account for this relationship. The present study extends these observations by focusing specifically on body-water characteristics. Additional sensitivity analysis incorporating FFMI showed that the positive association between TBW and retrospectively reported injury history remained similar after accounting for fat-free mass relative to height. This suggests that the association was not fully explained by differences in FFMI. Nevertheless, because TBW is closely related to fat-free tissue, adjustment for FFMI does not establish an independent physiological effect of body water, and residual confounding by other body-composition characteristics remains possible. Consistent with the previous multidimensional analysis, training-context characteristics showed weaker associations with injury phenotypes than body-composition characteristics [24]. Nevertheless, higher TBW should not be interpreted as causing greater reported injury occurrence; it may instead reflect broader body-composition or physiological characteristics associated with injury occurrence. Accordingly, TBW may be better viewed as an integrative body-composition-related characteristic rather than an isolated hydration-related risk factor.

The inverse association between ECW/TBW and retrospectively reported injury history provides a distinct perspective on the relationship between body-water characteristics and musculoskeletal injuries. Evidence directly relating fluid compartment distribution to injury is scarce, while previous studies have primarily examined ECW/TBW in relation to body composition, muscle characteristics, training status, or clinical conditions [27,28,29]. In generally healthy adults, ECW/TBW has been strongly associated with body-composition characteristics and has shown particularly strong discrimination for low muscle mass, suggesting that the ratio may capture information related to tissue composition rather than hydration status alone [29]. Studies in clinical populations further demonstrate that ECW/TBW is sensitive to changes in fluid distribution between intracellular and extracellular compartments [27,28], although these findings cannot be directly extrapolated to healthy, physically active individuals. Importantly, the opposing associations observed for TBW and ECW/TBW indicate that overall body water and its relative distribution should not be interpreted interchangeably. TBW reflects the overall water component of body mass, whereas ECW/TBW characterizes its relative compartmental distribution [29]. Thus, the two measures may capture different aspects of body composition and fluid organization that could show distinct relationships with injury occurrence. The additional FFMI-adjusted sensitivity analysis also showed that the inverse association between ECW/TBW and retrospectively reported injury history remained similar, suggesting that this relationship was not fully accounted for by differences in FFMI. However, this finding does not establish that ECW/TBW reflects a body-water-specific mechanism independent of broader body-composition characteristics. However, because TBW and ECW/TBW were examined in separate models, these findings do not establish whether either indicator provides an independent or incremental contribution beyond the other. However, because body water was measured at a single time point and injuries were reported for the preceding 12 months, the inverse ECW/TBW association cannot be interpreted as evidence of a protective effect and requires prospective confirmation.

The Bayesian approach provided additional information by quantifying posterior support for meaningfully negative, practically negligible, and meaningfully positive associations rather than relying solely on rejection of a null hypothesis. This distinction is particularly relevant in injury research, where uncertainty around an estimated association may reflect either evidence compatible with a practically negligible effect or insufficient information to distinguish among competing effect directions. Bayesian inference allows these possibilities to be expressed directly as probabilities assigned to predefined regions of the posterior distribution [30,31]. Posterior support was concentrated in the positive region for TBW and the negative region for ECW/TBW, with negligible support for the opposite directions. Thus, the findings do not merely indicate that the two body-water characteristics were associated with injury occurrence; they provide probabilistic evidence regarding both the direction and practical magnitude of these associations. These conclusions remained directionally consistent across alternative ROPE definitions, although the posterior probabilities assigned to the directional hypotheses varied with the width of the equivalence region [32]. Importantly, the ROPE thresholds used in this study represent operational definitions of practical equivalence rather than clinically validated cutoffs for musculoskeletal injury outcomes. In the absence of established minimum clinically important associations for TBW and ECW/TBW in relation to injury history, the sensitivity analyses provide an assessment of how the interpretation depends on the selected threshold, rather than confirmation of clinical significance. Accordingly, posterior probabilities should be interpreted in relation to the specified ROPE and should not be considered direct evidence of clinical importance. If replicated prospectively, body-water characteristics may contribute to the characterization of individual differences associated with musculoskeletal injury history, although their independent contributions and potential predictive relevance remain to be established.

The study combined complementary measures of body water with adjustment for physical activity and training exposure and a Bayesian framework allowing direct evaluation of competing directional hypotheses. Several methodological limitations warrant consideration. An important limitation is the cross-sectional design combined with retrospective assessment of musculoskeletal injuries over the preceding 12 months, which introduces the possibility of recall bias and prevents establishing temporal relationships. Because body-water characteristics were measured only once, it cannot be determined whether the observed TBW and ECW/TBW values reflected participants’ physiological status at the time of injury. Furthermore, reverse causation cannot be excluded, as previous injuries may have influenced subsequent training exposure, body composition, and fluid compartment distribution. Consequently, the observed associations should be interpreted as relationships between body-water characteristics measured at assessment and retrospectively reported injury history, rather than as evidence of prospective injury risk or causal effects. Bioelectrical impedance provides indirect estimates of body-water compartments, and these estimates may be influenced by short-term variations in hydration status. Although measurements were conducted within a standardized morning time window and participants received premeasurement instructions, compliance was not independently verified. Moreover, recent exercise, caffeine or alcohol consumption, and bladder status were not systematically documented, potentially introducing additional measurement variability. The adjusted models accounted for sex, total physical activity, weekly training volume, and training age. An additional sensitivity analysis incorporated FFMI to examine potential confounding by body composition. Although the estimated associations remained similar after this adjustment, FFMI does not capture all aspects of body composition, and the strong physiological relationship between total body water and fat-free tissue limits the interpretation of TBW as an independent body-water characteristic. Residual confounding by fat mass, sport type, training intensity, and other individual characteristics cannot be excluded. Residual confounding by sport type, training intensity, and other individual characteristics also cannot be excluded. Additionally, because TBW and ECW/TBW were analyzed in separate models, their independent and incremental contributions could not be evaluated. Future studies should examine their joint associations, with appropriate assessment of collinearity and model performance. Future studies should incorporate more comprehensive body-composition and sport-specific training measures to clarify whether body-water indicators provide information beyond these established characteristics. Finally, the sample consisted of physically active university students, limiting generalizability to other populations. Longitudinal studies incorporating repeated body-water measurements and prospective injury surveillance are needed to establish temporal direction and evaluate the physiological and potential predictive relevance of these associations.

## 5. Conclusions

Total body water and relative extracellular water distribution showed distinct and opposing associations with retrospectively reported musculoskeletal injury history in physically active young adults. Higher TBW showed a high posterior probability of a meaningfully positive association, whereas higher ECW/TBW showed a high posterior probability of a meaningfully negative association. These findings remained directionally consistent after adjustment for sex, physical activity, and training exposure and across alternative definitions of practical equivalence. Overall body water and its relative compartmental distribution may therefore represent distinct physiological characteristics associated with previous musculoskeletal injuries, although their independent or incremental contributions remain to be established.

These findings should be regarded as exploratory and hypothesis-generating. The cross-sectional design, retrospective assessment of injuries, and limited discriminatory performance of the models preclude conclusions regarding temporal direction, causality, or prospective injury prediction. Accordingly, the results do not support the immediate use of TBW or ECW/TBW for clinical screening or individual injury-risk classification. Longitudinal studies incorporating repeated body-water assessments, prospective injury surveillance, and external validation are needed to clarify the physiological basis and potential clinical relevance of these associations.

## Figures and Tables

**Table 1:** General characteristics of the study participants (N = 418).

Variable			Males *n* = 199			Females *n* = 219			
	Mean	95%CI Lower	95%CI Upper	SD	Mean	95%CI Lower	95%CI Upper	SD	*p*
Age [y]	20.73	20.61	20.85	0.85	20.56	20.46	20.65	0.74	**0.025**
Body height [cm]	182.19	181.20	183.18	7.10	168.17	167.37	168.97	6.01	**<0.001**
Body weight [kg]	79.63	78.25	81.01	9.87	60.86	59.65	62.06	9.05	**<0.001**
BMI [kg/m^2^]	23.97	23.62	24.32	2.48	21.49	21.12	21.85	2.71	**<0.001**
FFMI [kg/m^2^]	19.95	19.70	20.19	1.74	16.37	16.17	16.57	1.49	**<0.001**
TBW [% body mass}	54.71	53.61	55.82	7.91	45.30	43.76	46.83	11.50	**<0.001**
ECW/TBW [%}	37.40	37.12	37.68	2.00	38.68	38.39	38.98	2.20	**<0.001**
ICW/TBW [%]	62.63	62.35	62.91	2.03	61.33	61.03	61.63	2.24	**<0.001**
PA [MET/min/week]	3608.2	3418.5	3797.9	1356.9	3019.8	2886.5	3153.1	1000.9	**<0.001**
TV [h·week^−1^]	6.14	5.60	6.68	3.88	5.66	5.15	6.17	3.82	0.200
TA [years]	3.61	3.43	3.79	1.29	3.11	2.92	3.31	1.47	**<0.001**

Abbreviations: BMI—body mass index; FFMI—free fat mass index, TBW—total body water, ICW/TBW—intracellular water as a percentage of total body water; ECW/TBW—extracellular water as a percentage of total body water, PA—physical activity calculated based on the International Physical Activity Questionnaire (IPAQ); TV—training weekly volume (expressed in hours per week); TA—training age (experience expressed in years); SD—standard deviation; CI—confidence interval. Statistically significant values are highlighted in bold.

**Table 2:** Bayesian logistic regression analysis of injury occurrence.

Predictor	Model	β (95% CrI)	OR (95% CrI)	P(H−)	P(H0)	P(H+)
TBW	Unadjusted	0.346 (0.153–0.545)	1.414 (1.165–1.725)	0.000	0.006	**0.994**
TBW	Adjusted	0.324 (0.104–0.545)	1.383 (1.110–1.725)	<0.001	0.022	**0.978**
ECW/TBW	Unadjusted	−0.271 (−0.471–−0.079)	0.763 (0.625–0.924)	**0.958**	0.042	0.000
ECW/TBW	Adjusted	−0.268 (−0.483–−0.055)	0.765 (0.617–0.947)	**0.936**	0.064	<0.001

Note: Continuous variables associated with injury history were standardized. ORs therefore represent the change in odds of reporting ≥1 injury associated with a 1-SD increase in the respective body water indicator. H−: β < −0.10; H0: −0.10 ≤ β ≤ 0.10; H+: β > 0.10. Adjusted models included sex, total physical activity, weekly training volume, and training age. Statistically significant values are highlighted in bold.

## Data Availability

The data presented in this study are available on request from the author.

## Supplementary Materials

The following supporting information can be downloaded at: https://www.mdpi.com/article/10.3390/jcm15218392/s1, Table S1: Convergence and sampling diagnostics for the Bayesian logistic regression models; Table S2: Sensitivity of posterior hypothesis probabilities to alternative ROPE definitions; Table S3: Sensitivity of Bayesian regression estimates and posterior hypothesis probabilities to alternative prior specifications; Table S4: Sensitivity analyses adjusted for FFMI; Table S5: Comparison of separate and mutually adjusted Bayesian logistic regression models for total body water and extracellular-to-total body water ratio in relation to retrospectively reported musculoskeletal injury history.
