# Supplementary material for: Total Body Water and Fluid Distribution in Relation to Musculoskeletal Injury History: A Bayesian Analysis in Physically Active Young Adults
Source: J Clin Med. 2026 Oct 28;15(21):8392. doi: 10.3390/jcm15218392 (PMC13763773; doi:10.3390/jcm15218392)
Supplement: Supplementary file 1 [file jcm-15-08392-s001.zip › Supplementary Table S5. Comparison of separate and mutually.pdf]

Supplementary Table S5. Comparison of separate and mutually adjusted Bayesian logistic regression models for total body water and extracellular-to-total body water ratio in relation to retrospectively reported musculoskeletal injury history.

**Model**	**Predictor**	**β (95% CrI)**	**P(H−)**	**P(H0)**	**P(H+)**
Separate TBW	TBW	0.324 (0.101–0.540)	0.01%	2.43%	97.57%
Separate ECW/TBW	ECW/TBW	−0.268 (−0.480 to −0.057)	94.12%	5.86%	0.03%
Joint model	TBW	0.308 (−0.095 to 0.718)	2.41%	13.53%	84.07%
Joint model	ECW/TBW	−0.021 (−0.410 to 0.372)	34.56%	38.26%	27.18%

Footnote: All models were adjusted for sex, total physical activity, weekly training volume, and training age. Continuous predictors were standardized. β represents the posterior mean of the log-odds coefficient, and CrI denotes the 95% credible interval. H−, H0, and H+ correspond to coefficients below −0.10, within ±0.10, and above 0.10, respectively. The supplementary joint model included TBW and ECW/TBW simultaneously.
