# Supplementary material for: Total Body Water and Fluid Distribution in Relation to Musculoskeletal Injury History: A Bayesian Analysis in Physically Active Young Adults
Source: J Clin Med. 2026 Oct 28;15(21):8392. doi: 10.3390/jcm15218392 (PMC13763773; doi:10.3390/jcm15218392)
Supplement: Supplementary file 1 [file jcm-15-08392-s001.zip › Supplementary Table S1. Convergence and sampling diagnostics.pdf]

Supplementary Table S1. Convergence and sampling diagnostics for the Bayesian logistic regression models.

Model	Max R-hat	Min bulk ESS*	Min tail ESS*
TBW – Unadjusted	1.0019	3210	4573
TBW – Adjusted	1.0015	12000	7452
ECW/TBW – Unadjusted	1.0017	2652	3840
ECW/TBW – Adjusted	1.0008	12000	6811

Note: R-hat, potential scale reduction factor; ESS, effective sample size. Diagnostics were obtained from an independent Python MCMC analysis; ESS values are approximate.
