# Supplementary material for: Total Body Water and Fluid Distribution in Relation to Musculoskeletal Injury History: A Bayesian Analysis in Physically Active Young Adults
Source: J Clin Med. 2026 Oct 28;15(21):8392. doi: 10.3390/jcm15218392 (PMC13763773; doi:10.3390/jcm15218392)
Supplement: Supplementary file 1 [file jcm-15-08392-s001.zip › Supplementary Table S2. Sensitivity of posterior hypothesis probabilities.pdf]

Supplementary Table S2. Sensitivity of posterior hypothesis probabilities to alternative ROPE definitions.

**Predictor**	**Model**	**ROPE**	**P(H−), %**	**P(H0), %**	**P(H+), %**
TBW	Unadjusted	±0.05	0.0	0.1	99.9
		±0.10	0.0	0.6	99.4
		±0.20	0.0	7.1	92.9
TBW	Adjusted	±0.05	0.0	0.6	99.4
		±0.10	0.0	2.2	97.8
		±0.20	0.0	13.6	86.4
ECW/TBW	Unadjusted	±0.05	98.7	1.2	0.0
		±0.10	95.8	4.2	0.0
		±0.20	76.0	24.0	0.0
ECW/TBW	Adjusted	±0.05	97.7	2.2	0.1
		±0.10	93.6	6.4	0.0
		±0.20	73.1	26.9	0.0
