# Supplementary material for: Total Body Water and Fluid Distribution in Relation to Musculoskeletal Injury History: A Bayesian Analysis in Physically Active Young Adults
Source: J Clin Med. 2026 Oct 28;15(21):8392. doi: 10.3390/jcm15218392 (PMC13763773; doi:10.3390/jcm15218392)
Supplement: Supplementary file 1 [file jcm-15-08392-s001.zip › Supplementary Table S3. Sensitivity of Bayesian regression.pdf]

Supplementary Table S3. Sensitivity of Bayesian regression estimates and posterior hypothesis probabilities to alternative prior specifications.

Model	Prior β	β (95% CrI)	P(H−), %	P(H0), %	P(H+), %
TBW unadjusted	N(0, 0.5)	0.338 (0.146–0.534)	0.0	0.8	99.2
	N(0, 1)	0.348 (0.153–0.547)	0.0	0.6	99.4
	N(0, 2)	0.351 (0.155–0.551)	0.0	0.6	99.4
TBW adjusted	N(0, 0.5)	0.317 (0.103–0.532)	0.0	2.3	97.7
	N(0, 1)	0.324 (0.104–0.546)	0.0	2.3	97.7
	N(0, 2)	0.326 (0.104–0.550)	0.0	2.3	97.7
ECW/TBW unadjusted	N(0, 0.5)	−0.262 (−0.457 to −0.070)	95.1	4.9	0.0
	N(0, 1)	−0.270 (−0.468 to −0.075)	95.6	4.4	0.0
	N(0, 2)	−0.272 (−0.471 to −0.076)	95.7	4.3	0.0
ECW/TBW adjusted	N(0, 0.5)	−0.261 (−0.470 to −0.055)	93.7	6.3	0.0
	N(0, 1)	−0.268 (−0.481 to −0.057)	94.0	6.0	0.0
	N(0, 2)	−0.269 (−0.484 to −0.057)	94.1	5.9	0.0

Note: N(0, SD) denotes a normal prior with mean zero and the specified standard deviation for regression coefficients. The intercept prior was held constant at Normal(0, 1.5). Posterior probabilities were evaluated using the reference ROPE of ±0.10. Estimates were obtained using independent Laplace-based importance sampling and should be distinguished from the original brms MCMC estimates.
