# Supplementary material for: Total Body Water and Fluid Distribution in Relation to Musculoskeletal Injury History: A Bayesian Analysis in Physically Active Young Adults
Source: J Clin Med. 2026 Oct 28;15(21):8392. doi: 10.3390/jcm15218392 (PMC13763773; doi:10.3390/jcm15218392)
Supplement: Supplementary file 1 [file jcm-15-08392-s001.zip › Supplementary Table S4. Sensitivity analysis adjusted for FFMI.pdf]

Supplementary Table S4. Sensitivity analyses adjusted for FFMI.

**Model i predyktor**	**β (95% CrI)**	**OR (95% CrI)**	**P(H−)**	**P(H0)**	**P(H+)**
TBW, adjusted	0.319 (0.099–0.537)	1.375 (1.104–1.711)	0.0%	2.5%	97.5%
TBW + FFMI: TBW	0.306 (0.087–0.527)	1.359 (1.090–1.693)	0.0%	3.3%	96.7%
TBW + FFMI: FFMI	0.245 (−0.046–0.536)	1.278 (0.955–1.709)	1.0%	15.3%	83.7%
ECW/TBW, adjusted	−0.263 (−0.473 to −0.053)	0.769 (0.623–0.948)	93.6%	6.4%	0.0%
ECW/TBW + FFMI: ECW/TBW	−0.279 (−0.491 to −0.068)	0.756 (0.612–0.934)	95.1%	4.8%	0.0%
ECW/TBW + FFMI: FFMI	0.301 (0.011–0.592)	1.351 (1.011–1.807)	0.3%	8.4%	91.3%
